# Rationale and design of “Hearts & Parks”: study protocol for a pragmatic randomized clinical trial of an integrated clinic-community intervention to treat pediatric obesity

**DOI:** 10.1186/s12887-020-02190-x

**Published:** 2020-06-26

**Authors:** Sarah C. Armstrong, McAllister Windom, Nathan A. Bihlmeyer, Jennifer S. Li, Svati H. Shah, Mary Story, Nancy Zucker, William E. Kraus, Neha Pagidipati, Eric Peterson, Charlene Wong, Manuela Wiedemeier, Lauren Sibley, Samuel I. Berchuck, Peter Merrill, Alexandra Zizzi, Charles Sarria, Holly K. Dressman, John F. Rawls, Asheley C. Skinner

**Affiliations:** 1 Department of Pediatrics, Duke University, Durham, NC 27710 USA; 2 Duke Clinical Research Institute, Duke University, Durham, NC 27710 USA; 3 Duke Molecular Physiology Institute, Duke University, Durham, NC 27710 USA; 4 Department of Medicine, Duke University, Durham, NC 27710 USA; 5 Department of Family Medicine and Community Health, Duke University, Durham, NC 27710 USA; 6 Department of Psychiatry and Behavioral Sciences, Duke University, Durham, NC 27710 USA; 7 University of North Carolina School of Medicine, Chapel Hill, NC 27516 USA; 8 Department of Statistical Science, Duke University, Durham, NC 27710 USA; 9 Department of Molecular Genetics and Microbiology, Duke University, Durham, NC 27708 USA; 10 Department of Population Health Sciences, Duke University, 215 Morris Street, Suite 210, Durham, NC 27701 USA

**Keywords:** Pediatric, Obesity, Community, Children, Adolescents, Cardiovascular, Quality of life, Fitness, Parks and recreation, Partnership

## Abstract

**Background:**

The prevalence of child and adolescent obesity and severe obesity continues to increase despite decades of policy and research aimed at prevention. Obesity strongly predicts cardiovascular and metabolic disease risk; both begin in childhood. Children who receive intensive behavioral interventions can reduce body mass index (BMI) and reverse disease risk. However, delivering these interventions with fidelity at scale remains a challenge. Clinic-community partnerships offer a promising strategy to provide high-quality clinical care and deliver behavioral treatment in local park and recreation settings. The Hearts & Parks study has three broad objectives: (1) evaluate the effectiveness of the clinic-community model for the treatment of child obesity, (2) define microbiome and metabolomic signatures of obesity and response to lifestyle change, and (3) inform the implementation of similar models in clinical systems.

**Methods:**

Methods are designed for a pragmatic randomized, controlled clinical trial (*n* = 270) to test the effectiveness of an integrated clinic-community child obesity intervention as compared with usual care. We are powered to detect a difference in body mass index (BMI) between groups at 6 months, with follow up to 12 months. Secondary outcomes include changes in biomarkers for cardiovascular disease, psychosocial risk, and quality of life. Through collection of biospecimens (serum and stool), additional exploratory outcomes include microbiome and metabolomics biomarkers of response to lifestyle modification.

**Discussion:**

We present the study design, enrollment strategy, and intervention details for a randomized clinical trial to measure the effectiveness of a clinic-community child obesity treatment intervention. This study will inform a critical area in child obesity and cardiovascular risk research—defining outcomes, implementation feasibility, and identifying potential molecular mechanisms of treatment response.

**Clinical trial registration:**

NCT03339440.

## Background

Cardiovascular disease begins in youth, with obesity as a significant risk factor [1, 2]. Emphasizing the critical need for early prevention and treatment, adult obesity often originates in childhood, and most children with obesity will become adults with obesity. In fact, one in three children in the US are overweight or obese, leading to significant risk for future cardiovascular disease [3]. The American Academy of Pediatrics and the US Preventative Service Task Force (USPSTF) recommend that pediatric providers screen all children aged 6–18 years for obesity annually, using the Centers for Disease Control and Prevention (CDC) sex- and age-specific BMI curves. Additionally, children with BMI at or above the 95th percentile should be referred to a comprehensive behavioral intervention of medium to high intensity, defined as achieving ≥26 h of contact over 6 months [4, 5]. The current recommendation has not met the needs of a diverse population of youth with obesity. Among low-income, racially diverse, and ethnically diverse populations, clinical treatment has not met this recommendation, and has not led to significant reductions in child weight [6, 7].

One central challenge for clinical programs is meeting the recommendation for ≥26 h of contact in a 6-month period. Typical office visits last 15 min; therefore, this requirement amounts to nearly 4 clinic visits every week for the 6-month period. The epidemic currently affects 12.5 million children; current healthcare practices do not have the capacity to absorb this volume of additional visits [8]. Additionally, poor show rates and low family engagement are known barriers to delivering treatment [9]. From the family’s perspective, parents most commonly cite lack of time, cost of travel, and inability to miss work to attend multiple clinical visits, which are sometimes perceived as low-value [10, 11]. The World Health Organization has proposed a new chronic disease model that links healthcare and community settings. In this model, clinic visits serve to screen and treat co-morbid health conditions of obesity, while community centers offer locally-accessible activities during evenings and on weekends engaging the whole family and supporting social interaction.

Prior interventional studies aimed at reducing child obesity have demonstrated a highly heterogenous response to treatment, often leading to no significant BMI reduction [12]. Within these studies there are individuals who are highly responsive to treatment, yet little is known about the factors that predict treatment response. The multi-omic data analysis, including interrogation of metabolite and microbiome features, holds tremendous promise to elucidate the underlying mechanisms and potential variability between individuals and their response to treatment [13]. However, to date, child obesity interventions are focused on clinical outcomes rather than biomarkers that may predict response to treatment. This significantly limits our ability to explain the variability seen in pediatric obesity clinical trials beyond demographics and psychosocial data. Additionally, prior interventional studies have not considered the implementation and dissemination strategy, limiting the replication and ultimate impact of the intervention at the population level. In order to move the field forward, it is critical to leverage interdisciplinary team science and bring together clinical, basic, and population science researchers.

In order to assess the effectiveness of a clinic-community collaboration to treat child obesity we are conducting a randomized controlled trial, called “Hearts and Parks.” This article describes the rationale, objectives, and initial protocol as of April 14, 2020 for the Hearts and Parks trial.

### Rationale for the clinic-community model

#### Clinical obesity treatment

Duke Children’s Healthy Lifestyles program represents the current standard of clinical care for pediatric obesity treatment. Patients meet with multiple providers (medical, nutrition, physical therapy, and mental health) at monthly intervals for 1 year or until the patient’s BMI is in a healthy range. All visits use Motivational Interviewing as a communication strategy [14]. The Healthy Lifestyles clinic is high-volume; 800 new patients aged 2–18 are seen each year, and more than 15,000 children have received care to date. A retrospective case-control cohort study (*n* = 281) demonstrated that patients who complete at least 4 visits in 12 months had a small but significant reduction in zBMI (− 0.03 to − 0.19) [7]. Effectiveness is dose-responsive, and use of text messages leads to a greater number of clinic visits and thus a higher dose. We conducted a prospective, randomized controlled pilot study (*n* = 101) which compared standard care with standard care plus daily text messages, delivered to the parent’s mobile device. Subjects who received texts attended more clinic visits than those not receiving texts (4.3 vs. 1.2, *p* < 0.001) [15].

#### Engagement in community based programs

Bull City Fit is a fitness program delivered through a local Parks and Recreation Department that provides 6 days a week of high-intensity exercise, sports and games, cooking classes, gardening, and swimming. Each session lasts 2 h and is offered to the patient and his or her family members. We have demonstrated that this program is highly engaging among a low-income, racially diverse, and ethnically diverse group. Retrospective baseline data from prior participants of the program (*n* = 500) were supplemented by key informant interviews, focus groups, and surveys to assess participant demographics, attendance, and reasons for participation. Participants believed the program helped them get healthier (96%), attend clinical appointments (85%), and feel more confident exercising in public (72%). The most common reason cited for regular attendance was that peers “look like me” (82%) [16, 17]. High levels of participation in Bull City Fit lead to measureable changes in BMI. A retrospective cohort study (*n* = 271) demonstrated a wide range of attendance, resulting in a range of 10–105 h of participation over 6 months. Higher participation was associated with greater reduction in zBMI (− 0.03 among those participating 20 h or less versus − 0.1 among those participating 80 h or more, *p* = 0.02) [16, 18]. A pilot randomized controlled trial of the clinic-community model demonstrated significantly greater contact hours, as well as improvement in physical activity and quality of life [19].

### Measurement rationale

Although BMI is a central measurement for determining the effectiveness of pediatric obesity interventions, other potential benefits are much broader and deserve equal attention. For this study, we are including a variety of measures to capture these benefits, with particular emphasis on physical fitness, gut microbiome, and peripheral blood metabolites.

#### Physical fitness measures

Physical fitness is often overlooked as a key outcome in child obesity trials, yet fitness confers many cardiovascular health benefits even in the absence of weight loss. Based on the initial work of Blair and colleagues in the late 1980s and early-to-mid 1990s [20, 21], and others following, it is clear that physical fitness is an important predictor of all-cause mortality and disease-free survival for a myriad of conditions; this is true for youth and adults. Recognition of this link is what drove the development of the President’s Council on Physical Fitness in the early 1960’s and the development of physical fitness programs in elementary, middle, and high schools. Furthermore, we know that it is beneficial to be physically fit for a whole host of human medical conditions. Consequently, a favorable outcome of an obesity management program for youth that involves exposure to regular physical activity is the acquisition of a training response manifested as an increase in physical fitness [22].

Vigorous aerobic physical activity, even if intermittent, improves child BMI. An after-school fitness program randomized youth (*n* = 206) with obesity to academic (sedentary) activities or 80 min of moderate-to-vigorous physical activity. Despite only 40% mean attendance, the intervention group demonstrated significant improvements in BMI, body fat percent, and cardiorespiratory fitness [23]. Additionally, child obesity can be reduced with vigorous activity rather than restriction of energy intake [22]. Finally, physical fitness confers both cardiovascular and metabolic health improvements [24].

#### Stool microbiome and metabolic byproducts

The emerging evidence for the intestinal microbiome and its metabolic byproducts as key mediators in the development of obesity and response to treatment is promising. Previously, human observational studies and stool transplantation experiments in animal models (largely focused on adults) using high throughput molecular profiling platforms have found novel interconnections among obesity, insulin resistance, metabolic pathways, inflammation, and intestinal microbiota [25–32]. Recent observational studies in humans and animal models have also established that exercise can influence intestinal microbiota composition with increased efficacy during early life stages, though the consequences on microbiota function are unknown [33]. Studies in adults have also established that dysregulated metabolic pathways can be beneficially modified by exercise [34]. Children with severe obesity mimic adult phenotypes regarding metabolic and cardiovascular risk [35–38]. However, unlike adults, these children are at the earliest stages of disease, have fewer and less severe co-morbid conditions, and tend to be treatment-naïve. For these reasons, children with obesity present a unique opportunity and an ideal population in which to garner deeper insights into the obesity-associated microbiome and related metabolic pathways.

### Objectives

The Hearts & Parks study has three broad objectives: (1) evaluate the effectiveness of the clinic-community model for the treatment of child obesity, (2) define microbiome and metabolomic features associated with child obesity and intervention outcomes, and (3) inform the implementation of similar models in clinical systems.

## Methods/design

The Hearts & Parks trial, https://www.clinicaltrials.gov/ct2/show/NCT03339440, is a randomized controlled effectiveness trial to evaluate *Objective 1*: the integrated clinic-community approach to child obesity treatment compared with routine primary care. The hypothesis is tested through three aims. *Aim 1:* To evaluate the effectiveness of an integrated, clinic-community intervention to reduce child BMI at 6 months as compared with standard of care. We **hypothesize** that (1a) Bull City Fit will lead to a greater 6-month reduction in child BMI than standard care, and (1b) Sustained improvements at 1 year. *Aim 2:* To evaluate the effectiveness of an integrated, clinic-community intervention to improve cardiovascular health at 6 months as compared with standard of care. We hypothesize that Bull City Fit will lead to a greater 6-month improvement in (2a) peak heart rate during a cardiorespiratory fitness challenge and (2b) blood pressure and blood lipids, as compared with standard care. Finally, we include several secondary and exploratory outcomes, focused on social and emotional health, parent outcomes, and sleep.

Utilizing the subjects enrolled in the Hearts & Parks trial, we address *Objective 2*: examining microbiome and metabolic pathways. These aims include (1) identify associations that will inform the discovery of novel microbiota-related molecular pathways associated with obesity and cardiovascular fitness; (2) identify biomarkers that predict successful BMI reduction and exercise intervention efficacy in children and adolescents with obesity; (3) determine whether microbiome related molecular pathways are modifiable through programs of BMI reduction and exercise; and (4) determine how these pathways and biomarkers change as a function of life stage and disease status across the lifespan.

To address *Objective 3*, informing the implementation of the intervention in clinical and community systems, we use the Reach, Effectiveness, Adoption, Implementation and Maintenance (RE-AIM) [39] framework to measure and report facilitators and barriers to patient engagement, retention, successful outcomes, and project sustainability.

### Study design

**Fig. 1:**
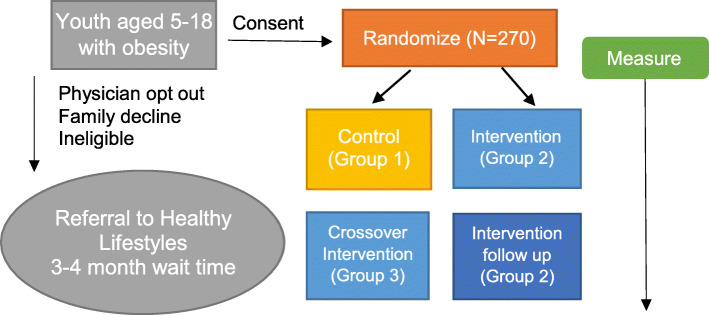
Hearts and Parks Study Diagram

The objectives above will be achieved through the completion of a prospective, randomized clinical trial as described in detail below. The study flow diagram (Fig. 1) highlights the recruitment, enrollment, randomization, and assessments for the study. We will use a two-arm, randomized crossover controlled trial to compare routine primary care management of childhood obesity versus a novel clinic-community partnership program to treat childhood obesity. This design will allow for a randomized comparison of Group 1 (6-month standard control) to Group 2 (immediate 6-month intervention program) and to Group 3 (delayed crossover intervention on the standard control group). The design will also allow us to observe intervention effects at 3 months in both intervention groups (Groups 2 and 3), and to track Group 2 for 6 additional months after active intervention to assess whether health benefits seen at 6 months were sustained to 12 months.

### Population

#### Study population

The intervention catchment area includes a 20-mile radius from the Edison Johnson Recreation Center. The majority of this radius is in Durham County, NC. The population is diverse: 42.2% non-Hispanic White, 38.6% African American, and 13.4% Hispanic. Approximately 62% of the population is below 185% of the federal poverty line. 66.2% of students in the Durham public schools over the 2016–2017 school year qualified for free and reduced lunch [40]. Patients referred to Healthy Lifestyles are representative of this population; administrative records estimate that between 65 and 82% of patients are enrolled in State Children’s Health Insurance Program or Medicaid.

#### Study recruitment and enrollment

Based on Healthy Lifestyles visit data (800 new patients/year) and pilot study data (100 subjects 5–12 years of age recruited in 9 months), we estimate that we can conservatively screen 500 subjects and enroll 270 in a 2-year enrollment period. We continuously monitor recruitment to ensure equal distribution among age groups, and plan to apply a stratification scheme if groups become imbalanced. As of April 2020, we have enrolled 261 children, with recruitment continuing through June 2020.

Primary care providers will refer eligible subjects using a novel Best Practice Alert (BPA, Epic) automatically generated by pre-specified inclusion criteria, and confirmed by a trained research assistant (Table 1). The BPA “fires” during the patient’s annual visit, and the pediatrician can click “ok to contact” or “opt out,” and this response enters a call list for the research staff to schedule the study visit. If the patient agrees to attend the study visit, the research coordinators complete the consent process with parents and assent with children, as approved by the Duke Health Institutional Review Board.

**Table 1:** Inclusion, Exclusion, and Opt-out Criteria

Inclusion Criteria	
• Child age 5–18 years • Child body mass index ≥95th percentile • Parent can speak and read in English or Spanish • Parent ownership of a device that is able to receive and send text messages	
Exclusion Criteria	
• Live farther than a 20-mile radius from the Bull City Fit program site • Endogenous or genetic cause of obesity • Taking a medication that causes weight gain • Participation in a pediatric weight management program within 12 months • Parent or child significant health problem that would limit participation • Pregnancy in patients of child-bearing age	
Opt-out Criteria	
✓ Primary care physician opts patient out of study for reasons including: severe obesity (BMI > 160% of sex- and age-specific 95th percentile), urgent co-morbidities, parental unwillingness to be contacted by a research assistant, or at physician clinical judgment.	

#### Randomization

Patients who consent for participation will undergo randomization in a 1:1 ratio following the baseline visit. Randomization occurs using the Research Electronic Data Capture (REDCap) system. Research coordinators notify patients of their assigned group following the baseline visit. Given the nature of the intervention, it is not possible to blind patients or providers to treatment assignment; therefore, unblinded randomization will be conducted.

#### Biospecimen population

A cohort of subjects participating in the primary trial will be included in the biospecimen analysis. Stool and blood serum samples will be collected at baseline and after the 6-month clinic-community intervention. Baseline samples will be from: (1) 100 obese adolescents (12–18 year olds); (2) 50 lean adolescent age- and sex-matched controls (12–18 year olds); (3) 100 obese children (5–11 year olds); and (4) 50 lean age- and sex-matched control children (5–11 year olds). Children and adolescents with obesity will be recruited through the primary study procedures until target sample size is met. Lean children will be recruited at the time of their well child visit, from the primary care clinic co-located with Healthy Lifestyles. Follow-up samples will only be collected from obese individuals.

**Fig. 2:**
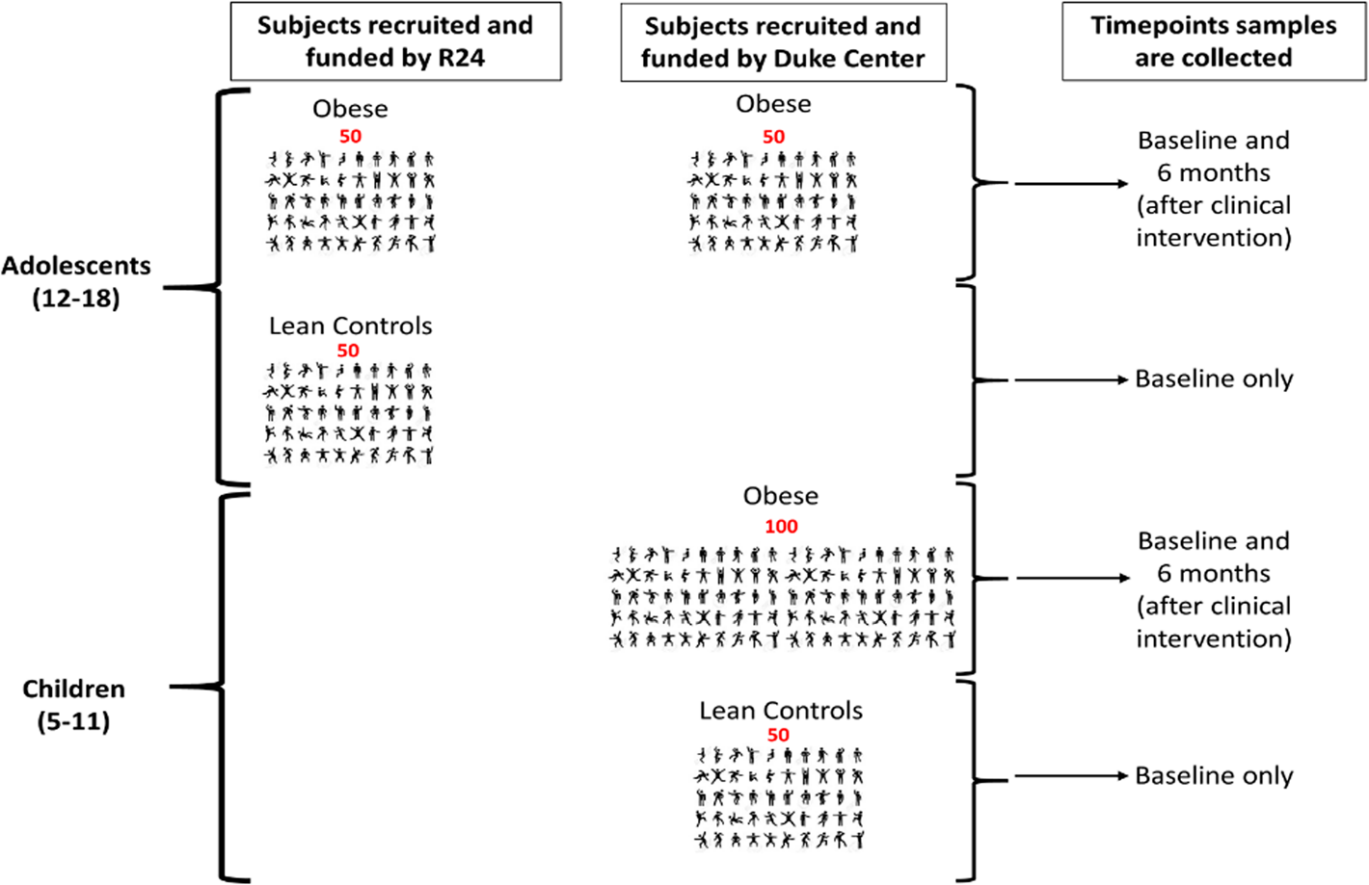
Overview of expansion of R24 Cohort by the newly funded Duke Center for Pediatric Obesity Research (American Heart Association Strategically Focused Research Network). Outline of which samples come from which cohort and age-group. Obese/lean is also listed. Only obese individuals will have follow-up samples collected

We will build upon and expand resources that already exist within Duke University, namely, a biorepository of stool and blood serum samples from participants with obesity, aged 12–18, which has been established by a NIDDK-funded R24 grant (R24-DK110492). Analyses for Objective 2 will be conducted on samples from the expanded, shared biorepository. Figure 2 outlines patient samples that will come from the R24 cohort, and those that will come from this project.

### Intervention

Subjects randomized to the intervention group enroll in the clinic-community intervention for 6 months. This includes Healthy Lifestyles clinic visits at baseline, 3-months, and 6-months, and twice-weekly sessions at the community center as described above. The clinic visits will include 30-min medical, dietary, and physical activity evaluation and counseling sessions. The sessions at the community center involve 30 min of warm up/check in time, at least 60 min of moderate-to-vigorous physical activity and 30 min of nutrition education. In total, subjects who participate fully will receive 4.5 h of clinical counseling, 52 h of physical activity, and 26 h of group-based nutrition counseling (108.5 h). In order to meet the recommended ≥26 h of contact over a 6-month period, subjects will need to participate in at least 25% of offered activities [5]. To enhance engagement and reinforce teaching, thrice-weekly text messages are sent to parent and child (if aged 11 or older, with parental permission) for the 6 months of the intervention. Within the community center, posters display playful characters designed to educate families about the experience and purpose of particular bodily sensations (e.g., Harold the Hunger Pain, Betty the Butterfly). These characters are further reinforced via text messages in which characters are reiterated, further information about that bodily sensation is provided, and information provided to increase interest in a variety of foods. Finally, monthly cartoon videos are designed to provide entertaining and accessible ways to put complex constructs like emotional eating and parent responsiveness into action.

The comparison group is an enhanced, 6-month wait list group. During the wait time, subjects will receive a non-obesity-related literacy intervention. This control intervention is consistent with Good Clinical Practice for the target population, as low-income families have fewer age-appropriate books per child in the home, and educational success is strongly mediated by literary skills [41, 42]. We will provide a $15 gift card each month to a locally owned book store in Durham. At the end of the 6-month wait time, subjects will be invited to participate in the intervention group.

### Measurement strategy

#### Trial measures

##### Body mass index:

The primary study outcome is BMI obtained through standardized measures of body weight and height using a digital scale and stadiometer [43]. Measures will be obtained at all data points. A large number of children referred to the healthy lifestyles clinic have a BMI significantly greater than the 95th percentile, and known challenges exist in measuring changes in BMI at extreme values. Therefore, as recommended by Flegal et al. we will assess and report child relative BMI, expressed as a standard deviation score (zBMI) and as a percent of the BMI value at the 95th percentile (%95th BMI) to evaluate and track obesity [44].

Cardiorespiratory Fitness is the principal secondary outcome, and will be assessed by heart rate at the completion of the YMCA submaximal bench-stepping test [45]. This was chosen over others due to its sensitivity to fitness-related changes in heart rate and ease of implementation in a mobile setting.

Given the complexity of pediatric obesity, a variety of secondary outcomes, including cardiovascular measures, social and emotional health, and behaviors, will also be assessed. Blood pressure will be measured with a calibrated auscultatory sphygmomanometer in the seated position with an appropriately sized cuff using standard methods [46]. Fasting blood sample for cardiometabolic biomarkers, including lipids, glucose, and transaminases will be obtained. Body fat percent will be measured using calibrated bioelectrical impedance. Parent BMI will be directly measured from parent height and weight using calibrated stadiometers.

Weight-specific quality of life will be measured using the validated “Sizing Me Up” child obesity-specific instrument, which has been used previously in the Healthy Lifestyles clinic population [47]. Research staff will administer survey-based instruments to the parent, child, or both to assess child temperament, mood, peer relationships, perceived family support, and body-esteem. We will employ measures developed by NIH PROMIS®, a patient-reported outcomes data management system to encourage more universal measurement of clinical outcomes across studies [48]. PROMIS is a data management system that allows researchers to choose independently validated items from domain-relevant item banks.

Child physical activity will be monitored by an activity tracker. Both intervention and control groups will receive activity trackers; recent evidence demonstrates that in the absence of coaching, an activity tracker alone does not impact body weight [49]. At the baseline visit, children will be provided with and taught how to use the activity tracker and to synchronize the data to the parent’s smartphone. Current guidelines recommend that children achieve 60 min of moderate-to-vigorous exercise, or 12,000 steps/day [50]. Subjects will wear the activity tracker continuously while in the study, and activity will automatically be collected, synced to the application by the participant, and downloaded daily by research assistants. The following data will be captured: steps/day and sleep. During participation in Bull City Fit, minutes/day of exercise and distance/day in miles will also be captured. We intentionally chose not to measure self-report dietary intake data because of the known poor internal and external validity of existing measures.

Multiple family characteristics are assessed as potential modifiers of intervention effectiveness, and to further characterize the population. Household income, transportation, parent stress, food insecurity, home food environment, and parental expectations for treatment will be collected by trained research staff at baseline using the instruments cited (Table 2). All parent measures will be available in English and Spanish.

**Table 2:** Measures and Schedule of Assessments

Measure	Description	0 m	3 m	6 m	9 m	12 m
*Child and Family Characteristics*						
Demographic	Race and ethnicity, household income, transportation	x				
Child nutrition and activity habits; self-report (screener)	FLASHE Food and Activity Screeners, Teen Version (Ages 10+) [51]	x		x		x
Parent nutrition and activity habits; self-report (screener)	FLASHE Food and Activity Screeners, Parent version [51]	x		x		x
Other characteristics	Parent stress [52], food insecurity [53], home food environment [54], and parental expectations for treatment [55]	x				
*Primary Outcome*						
Child BMI	Directly measured height and weight using calibrated scales	x	x	x	x	x
Child body fat percent	Measured via bioelectrical impedance (Tanita, TBF 300, Arlington Heights, Ill).	x	x	x	x	x
Child Waist Circumference (cm)	Standardized waist circumference measured by trained personnel, using cloth measuring tape, at level of umbilicus	x	x	x	x	x
*Secondary Outcomes*						
Child fitness	Baseline, 3-min, 4-min and 5-min heart rate after submaximal (3-min) bench-stepping test (YMCA)	x		x		x
Child physical activity; objectively tracked	Garmin VivoFit 3; every week sync; day “counts” if > 10 h/d + 4/7 d/wk.	Continuous				
Child blood pressure	Measured with calibrated auscultatory sphygmomanometer, supine position, appropriately sized cuff [56].	x		x		x
Fasting lipid profile	Obtained as per standard protocol in Healthy Lifestyles clinic at enrollment	x		x		x
Fasting glucose	Obtained as per standard protocol in Healthy Lifestyles clinic at enrollment	x		x		x
Alanine aminotransferase	Obtained as per standard protocol in Healthy Lifestyles clinic at enrollment	x		x		x
Child QOL	Sizing Me Up/Sizing Them Up [47] (< 10 parent/child together, 10+ child complete but with parent nearby)	x		x		x
Depression	PROMIS databank	x		x		x
Anxiety	PROMIS databank	x		x		x
Loneliness	PROMIS databank	x		x		x
Friendship	PROMIS databank	x		x		x
Parent-child relationship	PROMIS databank	x		x		x
Body appreciation	Body appreciation scale (13-item)	x		x		x
Parent BMI	Directly measured height and weight using calibrated scales	x		x		x
Sleep duration/ quality	Garmin VivoFit 3	x	x	x	x	x
*Implementation Measures*						
Adoption	BPA usage and referrals	Continuous				
Engagement	Program log; number of hours of “contact” with intervention (clinic+community).	Continuous				
Fidelity	SOFIT	Continuous				
*Basic Science Measures*						
Research Blood	Fasting serum samples	x		x		
Stool microbiome	16S rRNA gene sequencing	x		x		

#### Implementation measures

In addition to the above child and parent outcomes, we will include implementation measures [39]. Reach will be assessed by examining population participation in the intervention, including demographic representation. Adoption focuses on delivery from the primary care sites, including number of children referred through the BPA and subsequent enrollment rates. Engagement will be measured among intervention participants only, using number of total exposure hours to the intervention, including both clinic (Healthy Lifestyles) and community-based (Bull City Fit) activities. Clinic time will be recorded while the child is in clinic. A sign-in/sign-out system is used to track hours of participation at Bull City Fit. Fidelity, will be examined using the SOFIT assessment, which reports participation in the activities of the community program at a group level [57, 58], and child modifiers of participation, including social and emotional health. Maintenance will be assessed through continued engagement of primary care providers over time, and extrinsic factors that indicate likelihood of long-term sustainability, such as environmental and family influences.

### Trial study procedures

#### Data collection strategy

At the beginning of the intervention, all consented subjects (*n* = 270) have a baseline visit with a trained research assistant to collect clinical data, perform fitness testing and laboratory testing. Subjects are given an activity tracker to wear for the duration of the study. For those randomized into the intervention group, all measures for the study will be collected at routinely scheduled visits to the Healthy Lifestyles clinic at 3 months, 6 months, 9 months, and 12 months. Control subjects will have a study-only visit at baseline and at 3 months. At 6 months, control subjects will be crossed over to the intervention, and we will obtain measures at routinely scheduled Healthy Lifestyles clinic visits at 6 months, 9 months, and 12 months, which will correlate to intervention time points of baseline, 3 months, and 6 months. Clinical data are managed via REDCap, which provides a secure method of managing data to protect patient confidentiality. Surveys are completed directly in REDCap; additional are collected during study visits and as part of routine clinical care. Data are reviewed regularly for completeness. For biospecimens, participants are given a stool sample requisition kit, and either provide sample at the time of enrollment or produce sample at home. Samples are frozen immediately in a − 30 degrees (F) freezer (home or clinic), transported on dry ice, and then stored permanently at − 80 degrees (F). Serum samples for metabolomics are collected in the fasting state and immediately processed on site, and transported to the Duke Molecular Physiology Institute for further processing and storage.

#### Monitoring

We do not have a data monitoring committee, as the study is considered low risk. Because all children in the study are seen in routine clinical care, we do not have specific procedures for stopping the study or removing participants. These decisions are left to the discretion of their treating provider. As a low-risk study, we do not have planned interim analyses other than monitoring enrollment. Spontaneous adverse events are reporting in accordance with guidance from the Duke IRB.

### Biospecimen study procedures

To achieve the objectives of the biospecimen analyses, we will perform metabolomic, proteomic, and microbiomic profiling, along with stool transplants into gnotobiotic mice to test the impact of different microbiomes. For metabolomic profiling, we will use tandem flow injection mass spectrometry with addition of internal spiked standards for targeted profiling of 45 acylcarnitines and 15 amino acids [34, 59–63]. Proteomic profiling will be performed using 8 Olink targeted proteomics platforms (Cardiometabolic, Cell Regulation, CVD II, CVD III, Development, Immune Response, Inflammation, and Metabolism) giving us over 700 protein biomarkers [64–66]. For microbiome profiling, bacterial community composition in DNA isolated from stool samples will be characterized by amplification of the V4 variable region of the 16S rRNA gene by polymerase chain reaction using the forward primer 515 and reverse primer 806 following a version of the Earth Microbiome Project protocol. These primers (515F and 806R) carry unique barcodes that allow for multiplexed sequencing. Equimolar 16S rRNA PCR products from the samples will be quantified and pooled prior to sequencing. The pooled library will be submitted by the Duke Microbiome Shared Resource to the Duke Sequencing and Genomic Technologies shared resource for sequencing on a single lane of the Illumina MiSeq instrument configured for 250 base-pair paired-end sequencing runs. Microbiome bioinformatics will be performed with QIIME 22019.7 [67]. Raw sequence data will be demultiplexed and quality filtered using the q2-demux plugin followed by denoising with DADA2 (via q2-dada2) [68]. All amplicon sequence variants (ASVs) will be aligned with Mafft [69] (via q2-alignment) and used to construct a phylogeny with Raxml version 8 [70] (via q2-phylogeny). Taxonomy is assigned to ASVs using the q2-feature-classifier [71] classify-sklearn naïve Bayes taxonomy classifier against the SILVA 132 database [72].

Gnotobiotic mouse colonization will test if stool microbiome profiles from pediatric obesity patients are sufficient to phenocopy effects on host body weight and metabolic and proteomic profiles in gnotobiotic mice. Microbial communities of interest will be introduced by gavage into 12–16 week old germ-free male C57BL/6 J wild-type mice reared since weaning on a low-fat diet or moved at 6-weeks of age onto a high-fat diet. Transplanted low-fat fed and high-fat fed germ-free mice will be subsequently housed in hermetically-sealed isocages under gnotobiotic conditions [73]. Body weight will be measured at 0 and 14 days post-colonization. At baseline, 7 days, and 14 days post-colonization, plasma will be collected for metabolomic and proteomic analysis. At 14 days post colonization, animals will be euthanized and intestinal contents will be collected; liver, skeletal muscle and epididymal fat pads will be dissected, weighed, and flash frozen. Metabolomic and proteomic profiling will be performed on liver, muscle, and adipose tissue to determine the association between the candidate molecular pathways in key tissues.

Addressing potential barriers**.** We have conducted the preliminary studies to demonstrate both the feasibility of recruiting and retaining subjects using the proposed study design. However, there is the chance that we will have greater than the estimated 30% dropout. To minimize this, we will call and text families to keep them engaged. We will pair clinical and research blood sample collection where feasible. If we need to recruit more subjects, we will open additional clinical recruitment sites, including Lincoln Community Health Center, which serves many of Durham’s low-income families, or the two additional Duke Children’s Pediatric Clinics.

### Statistical analysis plan

#### Sample size

Preliminary data suggests that a meaningful difference in mean 6-month zBMI is − 0.10, and the pooled standard deviation across intervention groups is 0.2 [74]. We assume that 30% of subjects will be lost to follow-up prior to 6 months. Assuming normality of 6-month zBMI, power was computed using a two-sided t-test considering a type I error rate α = 0.05. In total, 270 subjects are needed to have 80% power to detect the meaningful difference in zBMI.

#### General analytic approach

To determine the effect of the intervention on 6 month change in zBMI, a generalized estimating equation model will be fit to estimate the difference in mean change in BMI between intervention groups, using an intention-to-treat approach [75]. This model will account for repeated observations of each child as well as correlation between siblings. A similar model will be used to assess the effect of the intervention on cardiorespiratory fitness as measured by heart rate at 3 min during the step test.

#### Statistical analysis of biospecimens

We will analyze four primary phenotypes: (1) obese vs. lean pediatric individuals (combining children and adolescents); (2) relative BMI as a continuous trait; (3) baseline cardiorespiratory fitness measures; and (4) obese vs. lean comparisons that are different in children vs. adolescents. Analyses will be corrected for multiple comparisons using False Discovery Rate (FDR) adjustment at the level of each individual platform or at the level of number of factors for the integrated ‘omics analyses. The results of these analyses will inform which patient stool samples will be used for gnotobiotic mouse experiments, and what traits to monitor in recipient gnotobiotic mice. We will also perform sensitivity analyses restricted to children and adolescents with clinical features similar to children who had the greatest relative BMI reduction. Power calculations for individual metabolites/proteins adjusted for a conservative correction for multiple comparisons at the level of factors estimated at 200 that will result from PCA analyses (α = 2.5 × 10–4). Based on effect sizes from previous studies, we have found a difference in mean (SD) levels between two groups of 0.32 (0.95) [76] as such, *N* = 109 in each group would provide 80% power. Thus, we will have sufficient power given this conservative estimate.

#### Implementation analysis

To determine the facilitators and barriers to Bull City Fit implementation and sustainability, we will use the RE-AIM framework [39], and define a priori successful targets for each factor. Enrollment of > 75% of eligible subjects will be considered successful reach. Effectiveness will be determined using the methods measuring the intervention effect on BMI and cardiorespiratory fitness described above. Adoption will be measured as the proportion of enrolled subjects that meet the minimum participation criteria of ≥26 h over the 6-month intervention. Implementation will be investigated using the distribution of intervention exposure over time among subjects in the intervention group. Additionally, the role of intrinsic (child-specific) factors on exposure will be investigated. Maintenance will be assessed in a similar manner as in the Implementation step; replacing the intrinsic factors with extrinsic ones (such as family and environment characteristics).

## Discussion

By increasing one’s lifetime risk for cardiovascular disease, the health consequences of childhood obesity are profound. The proposed research will directly address a critical gap in obesity treatment for children: we know that ≥26 h of treatment in a 6-month period is likely to be effective, yet we do not know how to deliver this care to diverse groups in a way that is effective and sustainable. We will combine the existing evidence for clinic and community-based interventions, tie them together with best-practice strategies for sustaining long-term partnerships, and use digital technology to maximize engagement. We propose to measure weight status and cardiorespiratory fitness along with multiple individual and environment-level factors to guide future implementation and sustainability. We will directly measure contact hours to determine if this intervention meets the current guidelines for child obesity treatment, addresses the barriers specific to a low-income and diverse population of high-risk youth, and demonstrates high potential for national dissemination and long-term sustainability. Through the basic science collaboration, we will gather samples to better understand metabolic and proteomic profiles associated with pediatric obesity as well as gut microbiota related molecular pathways in children as compared with adolescents. Our implementation component will inform the dissemination of successful aspects of the intervention through the broad network of municipal parks and recreation centers nationally.

## Data Availability

The datasets generated and/or analyzed during the current study are not publicly available as the study is ongoing. Following study completion, data will be available from the corresponding author upon reasonable request. Under a separate IRB-approved protocol (IRB Pro00074547), study participants consent for the long-term storage and future use of their data and biospecimens. This biorepository will be make publically available under a governance structure located at Duke University, with instructions for requesting samples here: https://sites.duke.edu/pomms/.

## Abbreviations

ASV: Amplicon Sequence Variant
BMI: Body Mass Index
BPA: Best Practice Alert
CDC: Centers for Disease Control and Prevention
USPSTF: US Preventative Service Task Force
